# The Effects of Six-Gram D-Aspartic Acid Supplementation on the Testosterone, Cortisol, and Hematological Responses of Male Boxers Subjected to 11 Days of Nocturnal Exposure to Normobaric Hypoxia

**DOI:** 10.3390/nu16010076

**Published:** 2023-12-25

**Authors:** Kamila Płoszczyca, Miłosz Czuba, Agnieszka Zakrzeska, Robert Gajda

**Affiliations:** 1 Department of Kinesiology, Institute of Sport, 01-982 Warsaw, Poland; 2 Faculty of Rehabilitation, Józef Piłsudski University of Physical Education in Warsaw, Marymoncka 34, 00-968 Warsaw, Poland; milosz.czuba@awf.edu.pl; 3 Department of Biotechnology, University of Medical Science in Bialystok, 15-875 Białystok, Polandgajda@gajdamed.pl (R.G.); 4 Center for Sports Cardiology, Gajda-Med Medical Center, 06-100 Pultusk, Poland

**Keywords:** DAA, testosterone, luteinizing hormone, hypoxia, altitude training, live high–train low, athletes

## Abstract

The aim of this study was to evaluate the effects of D-aspartic acid (DAA) supplementation during a simulated altitude protocol on the hormonal and hematological responses in athletes. We hypothesized that DAA supplementation would contribute to an increase in the luteinizing hormone (LH), free, and testosterone and a greater increase in hematological variables. Sixteen male boxers participated; they were randomly assigned to an experimental group (DAA) or a control group (C) and underwent 14 days of supplementation, 6 g/day of DAA. Both DAA and C participants were exposed to normobaric hypoxia (FiO_2_ = 15.5%; 2500 m) for 10–12 h a day over a period of 11 days. The results showed that DAA had no significant effect on resting, LH, or the testosterone/cortisol ratio during the training camp. Hypoxic exposure significantly (*p* < 0.05) increased red blood cell and reticulocyte counts as well as hemoglobin and hematocrit concentrations in both groups, but DAA had no significant effect on these changes. In conclusion, we found that DAA supplementation at a dose of 6 g/day for 14 days does not affect the testosterone, cortisol, or hematological responses of athletes during.

## 1. Introduction

Altitude/hypoxic training has been used for many years to improve exercise capacity in athletes. A number of concepts have been developed for using hypoxic conditions to improve performance in various sports. Popular methods include the live high–train low (LH–TL) protocol, where athletes live in hypoxic conditions at moderate altitudes (2000–2500 m above sea level, or a.s.l.) for 10–12 h a day (chiefly in the evening and at night) and train in normoxia, with normal oxygen availability [1]. Key benefits of the use of the LH–TL method include accelerated erythropoiesis and improved hematological indicators, which translate into an enhanced aerobic capacity in athletes [2,3]. Other adaptive changes observed in response to LH–TL include improved exercise economy [4,5] and improved muscle buffering capacity [6,7], which contribute to an increase in anaerobic performance in athletes [8].

The improvement in hematological variables expected from the use of LH–TL depends on many factors, including the duration of exposure to hypoxia and the intensity of the hypoxic stimulus, body iron stores in athletes, injuries and infections, and the anabolic–catabolic balance in the body [3]. Significant reductions in the testosterone/cortisol (T/C) ratio during altitude training may slow down erythropoiesis, especially in the early phase of acclimatization [9]. In a recent study [10], we observed a significant increase in blood testosterone (T) concentration during the LH–TL protocol, and we also demonstrated a positive correlation between blood T concentration in cyclists during the LH–TL protocol and increased blood hemoglobin concentration following hypoxic exposure.

Testosterone (T) has been proven to stimulate the hematopoietic system by increasing erythropoietin synthesis and secretion, acting on erythroid cells in the bone marrow, improving iron absorption and transport, stimulating iron incorporation into erythrocytes, increasing hemoglobin synthesis, prolonging erythrocyte survival time, and increasing 2,3-diphosphoglycerate (2,3-DPG) levels in red blood cells [11,12]. Increasing T concentrations in athletes during altitude/hypoxic training may potentially improve its effectiveness in adaptive hematological changes.

One legal substance being considered as an ergogenic aid to support T production is D-aspartic acid (DAA). D-aspartic acid (DAA) is a natural amino acid found in many tissues (including the brain, nervous system, endocrine glands, liver, ovaries, and testes), where it acts as a signaling factor [13]. D-aspartic acid affects the hypothalamic–pituitary–gonadal (HPG) axis by stimulating the secretion of gonadotropin-releasing hormones, which in turn leads to the increased release of luteinizing hormone (LH), which induces T production and release in the testes [13].

So far, few human studies have investigated the effects of DAA supplementation on T levels, and their findings have been inconsistent. Some studies showed that ingesting 3 g/day of DAA for a period of 14–28 days had no effect on blood levels of total and/or free testosterone (T and fT) in trained men [14,15,16,17,18]. However, Topo et al. [19] showed that DAA supplementation of 3 g/day for a period of 12 days increased the blood concentration of LH and T in young men by 33% and 42%, respectively, and the T levels remained heightened even three days after the end of supplementation. The above discrepancies are primarily attributed to variations in the baseline T levels among the subjects and differences in their levels of training.

To date, the scientific literature lacks sufficient reports on the use of DAA to promote hematological adaptations during altitude/hypoxic training in athletes. Hence, the present study aims to address this specific issue. Based on the above research results and theoretical underpinnings, this study investigated the following hypotheses: (1) DAA supplementation of 6 g/day would contribute to an increase in the blood concentration of LH, free testosterone (fT), and T in the participants; and (2) DAA supplementation during the LH–TL protocol results in a greater increase in the changes in hematological variables induced by hypoxic exposure.

## 2. Materials and Methods

### 2.1. Study Participants

This study involved 16 young men (aged 18 to 25 years) participating in competitive, Olympic-style boxing. The inclusion criteria required a minimum of a six-month washout period from previous altitude/hypoxic training and baseline blood testosterone levels within the age-specific reference range.

The participants were randomly assigned to the experimental group (DAA) and the control group (C) and underwent 14 days of supplementation. The DAA group (n = 8; age = 20.6 ± 2.1 years; height = 177.5 ± 1.7 cm; body mass = 75.1 ± 8.1 kg; % body fat = 10.5 ± 2.3; VO_2max_—56.9 ± 4.5 mL/kg/min) took 6 g/day of DAA, whereas the C group (n = 8; age = 19.1 ± 1.1 years; height = 181.4 ± 5.4 cm; body mass = 78.1 ± 6.7 kg; % body fat = 11.8 ± 3.3; VO_2max_—57.7 ± 2.5 mL/kg/min) received a placebo (cellulose). During the period of supplementation, all participants underwent LH–TL altitude training under normobaric hypoxia. The participants in the DAA and C groups were exposed to normobaric hypoxia for 10–12 h a day for a period of 11 days. All training sessions were conducted in normoxic conditions.

All participants had current valid medical examinations confirming they were in good health and could practice sports. Before commencing this study, all participants were informed about the purpose and course of this study and provided their written consent to participate. The athletes were also informed that they could withdraw from this study at any time without needing to provide a reason. Additionally, participants declared that for at least one month before testing, they had not taken any medications or dietary supplements. This study was approved by the ethics committee of the Institute of Sport—National Research Institute in Warsaw (No. KEBN-22-73-KP, 2 November 2022).

### 2.2. Study Design

**Figure 1:**
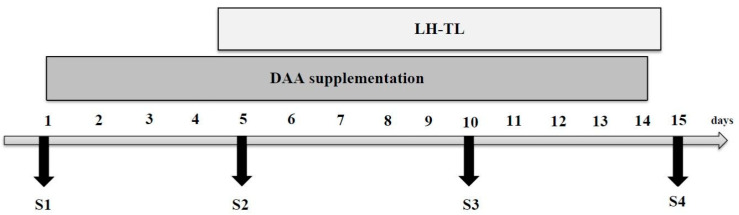
Study design. S1, S2, S3, S4—study series; LH–TL—live high–train low protocol; DAA—D-aspartic acid.

The experiment consisted of four study series (S1 to S4). S1 involved baseline tests before the start of supplementation and LH–TL training. S2 took place after the fourth day of supplementation and after the first night of exposure to hypoxia. S3 was conducted after the ninth day of supplementation and after the sixth night of the LH–TL protocol. Finally, S4 was performed immediately after the last night of the LH–TL protocol and the last day of supplementation. The study design is shown in Figure 1.

During each study series, venous blood samples (10 mL) collected from the basilic vein were obtained from the participants in the morning (7.30 a.m.) under fasting conditions. Two samples of venous blood were then collected at each time point on the 5th, 10th, and 15th day of the experiment, always after an active rest day. Of each pair of samples, one was drawn using an ethylenediaminetetraacetic (EDTA) tube (for morphology analysis); the other was drawn using an anti-coagulant tube and processed for serum for the other biochemical assays (T, fT, C, and LH). Creatine kinase (CK) activity was determined immediately after blood collection (Piccolo Express Chemistry Analyzer, Abaxis, Union City, CA, USA). After 30 min, blood samples were centrifuged at 1500× *g* for 15 min. The serum samples so obtained were stored at −70 °C until they were analyzed. The hematological markers (RBC, HGB, and HCT) were determined using a Sysmex XN 2000 analyzer (Sysmex Corporation, Kobe, Japan). Reticulocyte counts were measured manually. Serum levels of T, C, and LH were determined through the MCLIA method and the Cobas analyzer using Elecsys Testosterone II, Elecsys Cortisol II, and Elecsys LH tests (Roche Diagnostics GmbH, Mannheim, Germany). Levels of fT were evaluated through ELISA using a kit from Euroimmum.

### 2.3. DAA Supplementation

During this study, the participants in the DAA group took 6 g/day of DAA in the form of gelatin capsules for 14 days. The dose was divided into equal portions and administered twice a day at similar intervals. In turn, participants in the C group received a placebo (cellulose) in identical gelatin capsules, with the dose similarly divided into two equal portions. Participants were not aware of which substance they were ingesting. Supplementation began four days before the first exposure to hypoxia and continued throughout the whole of the LH–TL training period. D-aspartic acid (DAA) dose and supplementation time were selected based on the methodologies of previous studies regarding the ergogenic effects of DAA [14,15,16,17,18,19,20,21].

### 2.4. LH–TL Protocol and Conditioning Training

During this study, both DAA and C participants were subjected to continuous hypoxia for 10–12 h a day over a period of 11 days. In the rooms where the participants stayed in the evening and at night, the fraction of inspired oxygen (FiO_2_) in the gas mixture was 15.5%, which was equivalent to an altitude of approximately 2500 m a.s.l. This is an altitude that is recommended for the use of the LH–TL method in athletes to stimulate hematological changes [3]. The conditions of normobaric hypoxia were achieved using a specialized climate system (AirZone, AirSport, Miedzyzdroje, Poland). To ensure the safety of the participants during exposure to hypoxia, hemoglobin oxygen saturation and heart rate values were monitored using pulse oximeters every night.

The training program consisted of two basic microcycles (weeks) with progressively increasing loads, which were equal in both groups (Table 1). Intensity during all sessions was adjusted individually to each study participant based on the lactate threshold workload. Training sessions included specialized boxing drills and endurance as well as resistance exercises in both groups. The training load was recorded using heart monitors (Forerunner 245, Garmin, Olathe, KS, USA) and analyzed using Garmin Connect online platform (Garmin, Olathe, KS, USA).

### 2.5. Diet during the Experiment

As mentioned above, throughout the experiment, all athletes lived at the same accommodation and followed the same training schedule, sleeping time, and diet. During the experiments, the participants consumed a controlled mixed diet (50% CHO, 20% Fat, 30% Pro). Daily energy intake was set at 3500–4000 kcal (depending on the day), and the protein dose varied from 1.6 to 1.8 g/kg of body mass. During the experiment, the athletes consumed an isotonic sports drink or plain water. The dehydration level was assessed using Osmocheck calibrated in mOsm/kg H_2_O from 0 to 1500 mOsmols. Athletes did not take any nutritional supplements except DAA (experimental group—DAA) or a cellulose placebo (control group—C).

### 2.6. Statistical Analysis

The results of the experiment were analyzed using StatSoft Statistica 13.0 software. The results are presented as arithmetic means (x) with standard deviations (SD). The Lilliefors test was used to demonstrate the consistency of the results with normal distribution. The intergroup differences between consecutive research series were determined using a two-way ANOVA (group and training) with repeated measures. The significance of differences between individual research series (differences between series of testing) in the study groups was calculated using Tukey’s post hoc test. The area under the curve (AUC) for T and fT levels was calculated using the trapezoid method. Significant differences for AUC values between groups were determined using the one-way analysis of variance (ANOVA). The significance of differences between the study groups was calculated based on the post hoc Tukey’s test. The relationships between AUC for T and fT levels and changes in selected hematological variables were analyzed using the Pearson’s correlation coefficient. Statistical significance was set at *p* < 0.05.

## 3. Results

### 3.1. Hormonal Response

**Figure 2:**
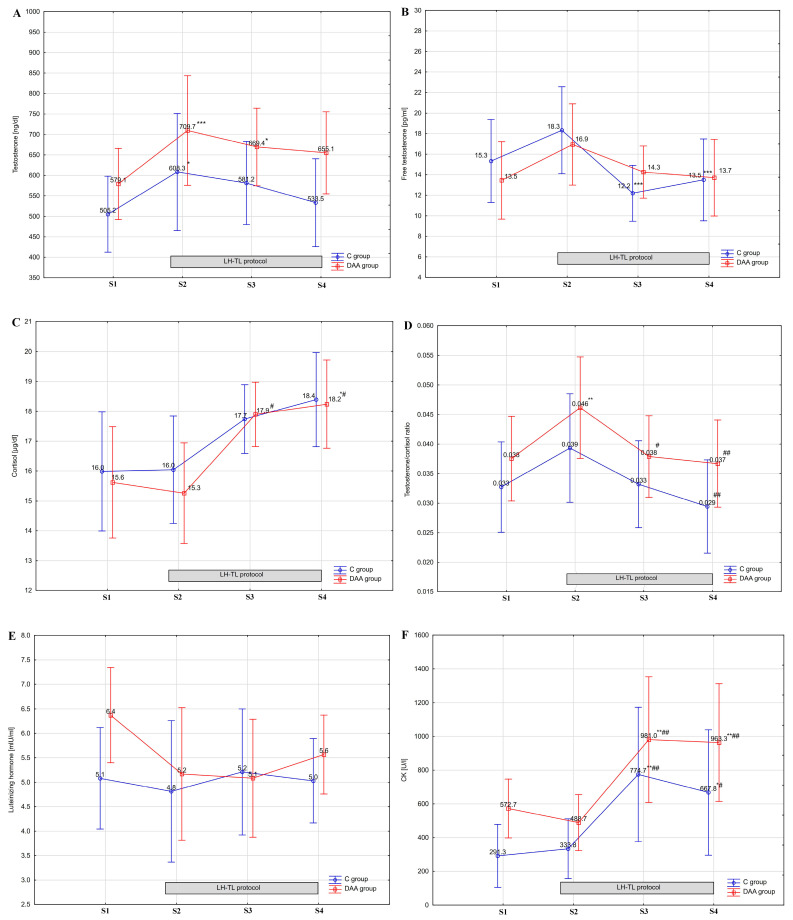
Blood serum testosterone (**A**), free testosterone (**B**), cortisol (**C**), testosterone/cortisol ratio (**D**), luteinizing hormone (**E**) levels, and creatine kinase—CK activity (**F**) in experimental (DAA) and control (**C**) groups during the experiment; *** *p* < 0.001, ** *p* < 0.01, * *p* < 0.05—statistically significant differences compared to baseline (S1); ## *p* < 0.01, # *p* < 0.05—statistically significant differences compared to S2. S1—First series of tests before the start of supplementation and LH–TL training; S2—Second series of tests after the 4th day of supplementation and after the 1st night of hypoxic exposure; S3—Third series of tests after the 9th day of supplementation and after the 6th night of the LH–TL protocol; S4—Fourth series of tests immediately after the last night (11th) of the LH–TL protocol and the last day (14th) of supplementation.

The analysis of variance (ANOVA) showed no statistically significant group × training interactions for changes in resting values of T, fT, C, or LH following DAA supplementation compared to the control group. However, ANOVA revealed a statistically significant effect of training on changes in T levels (F = 12.572, *p* < 0.001), fT levels (F = 12.529, *p* < 0.001), C levels (F = 11.230, *p* < 0.001), and T/C ratio (F = 13.973, *p* < 0.001) in the study groups. Exposure under hypoxic conditions caused a significant (*p* < 0.05) increase in T levels after the first night of the LH–TL protocol in the DAA and C groups (by 22.5% and 20.2%, respectively; Figure 2A) and after 6 nights of LH–TL, but only in the DAA group (by 15.6% compared to the baseline level) (Figure 2). During the experiment, there was a tendency towards decreasing values of fT concentration on the 10th and 15th day of the experiment (S3, S4); however, these changes were significant only in the C group (Figure 2B). The fT level significantly (*p* < 0.05) decreased in this group by 33.3% in S3 and 26.2% in S4 compared to the fT level in S2 (Figure 2B). The opposite tendency was observed in cortisol concentration in both groups. However, only changes on days 10 and 15 (S3, S4) in the DAA group were significant compared to the C levels observed on the 5th day of the experiment (S2) (Figure 2C). Additionally, a significant (*p* < 0.01) increase in the T/C ratio was observed only in the DAA group in S2. However, the T/C ratio values showed a significant decrease (*p* < 0.01) in S3 and S4 compared to the values observed in S2 in both groups (Figure 2D). There were no statistically significant differences in blood serum LH levels in any of the study groups (Figure 2E). In addition, the statistical analysis performed showed no significant differences in the AUC values for T, fT, LH, or C between the study groups (DAA vs. C; Table 2).

### 3.2. Hematological Response

The statistical analysis showed no significant group × training interactions for changes in resting values of red blood cell count (RBC), hemoglobin concentration (HGB), hematocrit (HCT), or blood reticulocyte percentage (Ret) following DAA supplementation compared to the control group (Table 2). However, ANOVA revealed a statistically significant effect of training on changes in RBC (F = 12.181, *p* < 0.001), HGB (F = 16.451, *p* < 0.001), HCT (F = 12.313, *p* < 0.001), and Ret levels (F = 47.186, *p* < 0.001) in the study groups. Tukey’s post hoc test showed a significant (*p* < 0.05) improvement in RBC, HGB, HCT, and Ret in both groups after the LH–TL protocol in S4 (Table 2). No significant correlations were found between the changes in T and fT levels, changes in AUC for T and fT, or changes in selected hematological variables.

### 3.3. Training Loads and Changes in Blood Creatine Kinase (CK) Activity

The statistical analysis showed no significant differences in training load (TSS; 2038 ± 54—DAA group vs. 2068 ± 43—C group) in the study groups. Additionally, ANOVA showed no statistically significant group × training interactions for changes in CK activity in the blood during the experiment. However, ANOVA revealed a statistically significant effect of training on changes in CK activity (F = 24.268 *p* < 0.001) in the study groups. Tukey’s post hoc test showed a significant (*p* < 0.05) increase in blood serum CK activity on the 10th and 15th days of the experiment (S3, S4) in both groups (DAA, C). Despite the lack of significant differences between groups regarding CK activity in S3 and S4, its levels were considerably above the normal range in both groups (Figure 2F).

## 4. Discussion

A high T blood concentration and a favorable T/C ratio are considered important factors influencing the effectiveness of altitude training [3]. Higher T levels not only increase protein synthesis and reduce protein breakdown but may also contribute to a higher rate and extent of hematological adaptive changes induced by altitude/hypoxic training. Testosterone is involved in the hormonal regulation of erythropoiesis [22]. Gonzalez et al. [23] found that high serum T levels were associated with excessive erythrocytosis in men at altitudes. In a recent study [10], our team found that in cyclists during 3-week training, the testosterone concentration and the T/C ratio were maintained at a higher level in a group that implemented the LH–TL protocol compared to a group staying in normoxia. Moreover, the T concentration during LH–TL training correlated with the magnitude of changes in HGB [10]. Therefore, in the current study, we sought to investigate whether the use of DAA, considered to be a potentially T-production-enhancing agent, would contribute during hypoxic training (LH–TL protocol) to an increase in T concentration in the blood and to an increase in hematological changes achieved in the subjects.

The results of our study showed that a 2-week DAA supplemental protocol (6 g per day) did not cause significant beneficial changes in blood testosterone (T, fT) concentration during LH–TL and did not affect the effectiveness of hypoxic exposure in terms of hematological changes in relation to the control group. We showed that in both groups, after the first night spent in hypoxic conditions (after 4 days of supplementation), there was an approx. 20% increase in T concentration in the blood. No significant changes in fT, LH, or C were noted. Changes in the T/C ratio were also observed. Initially, after 12 h of exposure to hypoxia (S2), there was a significant increase in T/C in the DAA group compared to the baseline studies. Notably, the changes in the C group had the same trend, but they were not statistically significant. The increase in the T/C ratio in the DAA group was temporary, and on the following days of the LH–TL protocol (S3 and S4), the T/C values no longer differed significantly from the baseline. In our opinion, the decrease in T/C values during S3 and S4 compared to the S2 measurement was due to the increasing fatigue of the subjects, as evidenced by significant changes in CK activity, as well as an increase in C concentration in the blood.

The observed increase in T concentration in the blood during S2 was the effect of 12 h of hypoxic exposure of the subjects, not the effect of the introduced DAA supplementation. Recently, Oyedokun et al. [24] concluded that acute hypoxic exposure promotes testosterone production mainly via stimulation of autophagy and upregulation of steroidogenic enzymes and the voltage-gated L-type calcium channel. These observations are corroborated by earlier reports of changes in testosterone concentrations under conditions of acute hypoxia [10,25]. In the present study, the application of DAA in the experimental group (for 4 days prior to exposure) did not affect the magnitude of the T response to acute hypoxia.

Our study results, indicating that DAA supplementation does not affect T or fT concentrations, are consistent with previous studies involving male athletes using various DAA doses (1.78 to 6 g per day) over different dosing periods (from 2 to 12 weeks) [14,15,16,17,18,21]. Luteinizing hormone (LH), one of the indicators of the HPG axis, also did not respond to DAA supplementation in our study, which is also in line with previous reports in this respect [14,15,16,18]. Crewther et al. [18] argue that one of the reasons for the lack of DAA efficacy could be the controlling mechanisms of the HPG axis, which may act to maintain endogenous T concentration at a certain level, potentially limiting the effect of supplementation. Alternatively, a simultaneous increase in aspartate oxidase (a catalyst for D-amino acids) may degrade DAA to limit the HPG axis response [14]. Interestingly, there are also reports indicating a decrease in T and fT concentrations in some resistance-trained men supplemented with DAA at a dose of 6 g per day for 2 weeks [16]. However, repeating this protocol over a 12-week training period did not result in any changes in T concentration [21]. The literature also includes some studies reporting an increase in T concentration in men supplemented with DAA; however, the subjects in these groups were characterized by low baseline T concentration in the blood [13,19,20]. The average baseline T concentrations in these studies (9–16 nmol/L) were lower than those observed in our present study (13.9–25.1 nmol/L—baseline), as well as in previous studies that did not confirm the beneficial effects of DAA (18–41 nmol/L) [14,15,16,17,18,21]. Willoughby and Leutholtz [14] have suggested that DAA may only be effective in men with reduced T concentrations.

As mentioned above, the ineffectiveness of DAA was previously explained by the autoregulatory control of the HPG axis, which may limit endogenous T production when it approaches or exceeds a certain individual threshold. The results of our study suggest that the autoregulatory control of the HPG axis depends on the strength of the stimulus. Our study found that after exposure to normobaric hypoxia, there was a significant increase in blood T concentration in the studied groups. The lack of change in T concentration after DAA supplementation, on the other hand, suggests that the stress caused by hypoxia seems to be a much stronger stimulus in this regard than the effect of DAA.

The only potentially beneficial change observed in our study resulting from DAA supplementation appears to be a significantly higher T concentration (by 15% vs. baseline) after 6 nights of the LH–TL protocol (after 9 days of supplementation), despite the accumulation of training loads. In the control group, the T concentration at this time tended to return to baseline values. Additionally, in the control group, on the 5th and 10th days of the experiment, the level of fT in the blood reached values lower than the baseline. In the DAA group, on the other hand, despite increasing fatigue over the consecutive days of training, the fT did not change. However, further analysis of the area under the curve (AUC) for T and fT concentrations in the blood during the entire intervention period did not show significant differences between the DAA and control groups. These findings allow us to conclude that the applied DAA supplementation did not cause significant beneficial changes in blood testosterone concentration during the given LH–TL protocol. In our opinion, the observed changes in the rate of return of T and fT concentrations to baseline values during training are most likely the result of the different reactivity of the subjects to hypoxic conditions and the imposed training loads rather than the effect of DAA.

As regards hematological changes, we observed a small but significant (*p* < 0.05) improvement in RBC, HGB, HCT, and Ret in both groups after 11 days of the LH–TL protocol. DAA supplementation did not cause major changes in selected hematological variables. The little improvement in hematologic variables is due to the relatively short duration of the LH–TL protocol. Larger changes in this area require longer exposure to hypoxia (usually 3–4 weeks) [3]. However, the observed changes are consistent with previous studies on the LH–TL protocol [2,10]. Unfortunately, in the present study, we did not confirm our previously observed correlations between blood T concentrations during the LH–TL protocol and increases in hemoglobin concentration, which may also be due to the shorter exposure to hypoxia.

## 5. Study Limitations

To the best of our knowledge, our study is the first in which the effect of DAA supplementation was assessed in normobaric hypoxia during the LH–TL protocol. Our experiment, however, is not without certain limitations. Firstly, we did not measure the levels of the analyzed indicators immediately before the initial exposure to hypoxia. An additional point of measurement would have allowed for a precise determination of whether there was a change in blood T levels after 4 days of DAA supplementation and whether the rise in T levels during S2 was due to hypoxia alone or the combined influence of DAA and hypoxia. However, logistical constraints (related to the athletes’ arrival at the training camp) prevented us from taking measurements on that particular day. Secondly, it would have been advantageous to determine the concentrations of DAA and D-aspartate oxidase in the blood. Such data would enable us to understand if oral DAA supplementation significantly alters the levels of DAA and D-aspartate oxidase in the blood. Future research should also consider a longer DAA supplementation period prior to altitude training and possibly using a 3 g/day dosage, as there are some indications that a 6 g/day dosage may lead to reduced T and fT concentrations [16].

## 6. Conclusions

Overall, we found that short-term DAA supplementation at a dose of 6 g/day for 14 days did not influence the testosterone, cortisol, or hematological responses of male boxers during hypoxic training (LH–TL protocol). The observed changes in blood T concentration after the first 12 h of exposure to hypoxia were, we conclude, the result of the strong effect of the hypoxic stimulus, not the result of the introduced supplementation. The transient maintenance of higher T levels and the inhibition of fT decrease during the LH–TL protocol in the supplemented group, in our opinion, is insufficient to state that DAA has a beneficial effect on testosterone response during hypoxic training. Moreover, contrary to our expectations, DAA supplementation also had no effect on the magnitude of hematological changes induced by hypoxic exposure. Therefore, we do not recommend DAA supplementation to improve hematological response during LH–TL training.

## Figures and Tables

**Table 1:** Training program during the experiment.

Day	Microcycle 1	Microcycle 2
1	Morning: First series of study (S1), Boxing training—technical training, boxing drills (60 min) Afternoon: Strength training—upper body (90 min)	Morning: Boxing training—high intensity (60 min) Afternoon: Strength training—lower body (90 min)
2	Morning: Boxing training—high intensity (60 min) Afternoon: Strength training—lower body (90 min)	Day off Active recovery
3	Morning: off Afternoon: Boxing training—high intensity (90 min)	Morning: Third series of study (S3), Endurance training—medium intensity (90 min) Afternoon: Boxing training—technical training, boxing drills (60 min)
4	Morning: Traveling Afternoon: Active recovery	Morning: Boxing training—high intensity (60 min) Afternoon: Strength training—upper body (90 min)
5	Morning: Second series of study (S2), Endurance training—medium intensity (90 min) Afternoon: Boxing training—technical training, boxing drills (60 min)	Morning: Endurance training—interval running—high intensity (60 min) Afternoon: Boxing training—technical training (60 min)
6	Morning: Boxing training—high intensity (60 min) Afternoon: Strength training—upper body (90 min)	Morning: Boxing training—high intensity (60 min) Afternoon: Strength training—lower body (90 min)
7	Morning: Endurance training—interval running—high intensity (60 min) Afternoon: Boxing training—technical training (60 min)	Day off Active recovery

**Table 2:** Selected hematological variables and area under the curve for testosterone (AUC T), free testosterone (AUC fT), cortisol (AUC C), and luteinizing hormone (AUC LH) during the experiment in DAA and C groups.

Variables	Group	(S1)	(S2)	(S3)	(S4)
RBC (mL/uL)	DAA C	4.90 ± 0.16 5.02 ± 0.47	4.90 ± 0.14 5.11 ± 0.51	4.95 ± 0.08 5.11 ± 0.43	5.09 ± 0.13 * 5.26 ± 0.48 *
HGB (g/dL)	DAA C	14.71 ± 0.81 14.96 ± 1.04	14.61 ± 0.71 15.08 ± 0.98	15.04 ± 0.68 15.22 ± 1.01	15.42 ± 0.56 * 15.70 ± 1.09 *
HCT (%)	DAA C	43.98 ± 1.91 44.25 ± 2.44	43.62 ± 1.89 44.10 ± 2.44	44.07 ± 1.59 44.73 ± 2.39	45.36 ± 1.27 * 45.79 ± 3.18 *
Ret(%)	DAA C	8.57 ± 4.27 10.05 ± 2.35	10.80 ± 3.69 11.96 ± 3.23	13.24 ± 2.89 ** 14.58 ± 3.39 ***	16.44 ± 4.06 *** 17.66 ± 3.36 ***
AUC T	DAA C	9981.4 ± 2398.5 8544.1 ± 1469.3			
AUC fT	DAA C	223.9 ± 44.2 224.6 ± 79.3			
AUC C	DAA C	250.4 ± 12.4 255.1 ± 28.3			
AUC LH	DAA C	81.1 ± 13.52 77.1 ± 23.4			

*** *p* < 0.001; ** *p* < 0.01; * *p* < 0.05 vs. baseline (S1). S1—First series of tests before the start of supplementation and LH–TL training; S2—Second series of tests after the 4th day of supplementation and after the 1st night of hypoxic exposure; S3—Third series of tests after the 9th day of supplementation and after the 6th night of the LH–TL protocol; S4—Fourth series of tests immediately after the last night (11th) of the LH–TL protocol and the last day (14th) of supplementation.

## Data Availability

The data presented in this study are available upon request from the corresponding author. The data are not publicly available due to privacy.
